# Protein-mRNA interactome capture: cartography of the mRNP landscape

**DOI:** 10.12688/f1000research.9404.1

**Published:** 2016-11-03

**Authors:** Sean P. Ryder

**Affiliations:** 1 Department of Biochemistry and Molecular Pharmacology, University of Massachusetts Medical School, Worcester, MA, 01605, USA

**Keywords:** RNA-binding proteins, RBPs, mRNA interactome discovery, messenger RNA

## Abstract

RNA-binding proteins play a variety of roles in cellular physiology. Some regulate mRNA processing, mRNA abundance, and translation efficiency. Some fight off invader RNA through small RNA-driven silencing pathways. Others sense foreign sequences in the form of double-stranded RNA and activate the innate immune response. Yet others, for example cytoplasmic aconitase, act as bi-functional proteins, processing metabolites in one conformation and regulating metabolic gene expression in another. Not all are involved in gene regulation. Some play structural roles, for example, connecting the translational machinery to the endoplasmic reticulum outer membrane. Despite their pervasive role and relative importance, it has remained difficult to identify new RNA-binding proteins in a systematic, unbiased way. A recent body of literature from several independent labs has defined robust, easily adaptable protocols for mRNA interactome discovery. In this review, I summarize the methods and review some of the intriguing findings from their application to a wide variety of biological systems.

## Introduction

Eukaryotic messenger RNAs (mRNAs) must be produced in the nucleus and then transported to the cytoplasm in order to function as a template for protein synthesis. This process is assisted at numerous levels by RNA-binding proteins (RBPs), many of which modify the fate of the mRNA ^1^. For example, alternative splicing or polyadenylation factors regulate mRNA processing during synthesis ^2– 5^. Some RBPs govern nuclear export and/or subsequent localization in the cytoplasm or nucleus ^6, 7^. Others regulate translation efficiency or mRNA decay ^8– 10^. Some might bind RNA with no meaningful biological outcome ^11^.

Despite RBPs’ regulatory potential, the identification of new ones remains a relatively slow process that typically requires focused, gene-specific studies. Few systematic approaches to identify new RBPs genome-wide are available. One approach relies on bioinformatics to search for hallmark domains and sequences that have been demonstrated to bind RNA in single gene studies ^12– 15^. Indeed, significant progress has been made using this approach to identify RBPs with important biological functions in a variety of experimental models. For example, the discovery that Pumilio binds to a critical sequence in the 3′ untranslated region (UTR) of *hunchback* mRNA in fly embryos ^16– 19^, together with the discovery that FBF binds to a similar sequence in the 3′ UTR of *fem-3* mRNA in *Caenorhabditis elegans* ^20, 21^, defined a new RBP domain termed “Puf” ( Pumilio- FBF). Homology searches subsequently led to the discovery of Puf-family RBPs in yeast, humans, worms, and several other species, many with important functions in translational control ^22– 29^. Such informatics-based approaches help to catalogue the RNA interactome but are necessarily limited by the requirement for prior knowledge that a given protein domain typically has RNA-binding activity.

Sometimes, such knowledge can be misleading. For example, the canonical RNA-recognition motif (RRM) does not always bind to RNA ^30^. Mago nashi (Mago) is a core component of the exon-junction complex deposited by the spliceosome upstream of splice junctions ^31, 32^. This protein contains an RRM and is present in a complex that binds to RNA. However, the RRM of Mago does not contact RNA directly. Instead, the crystal structure of *Drosophila melanogaster* Mago in complex with Y14 revealed that this protein makes use of the RRM domain to form an extended protein-protein interaction surface ^32, 33^. Several additional examples of protein-binding RRM domains have been identified since this surprising discovery ^30^.

Other approaches to map the RBP interactome are designed to identify all of the mRNAs that can bind to a single protein ^34– 39^ or all of the proteins that can bind to a given RNA sequence ^40, 41^. While both strategies are incredibly useful, until recently, no approach had been developed to identify all of the RBPs that touch mRNA in a cell or animal. A technological breakthrough from two independent labs has filled this gap ^42, 43^. Application of this technology by several labs is beginning to provide the first comprehensive glance at the RBP atlas in several systems ^44– 51^.

## The mRNA interactome in cultured mammalian cells

**Figure 1.**
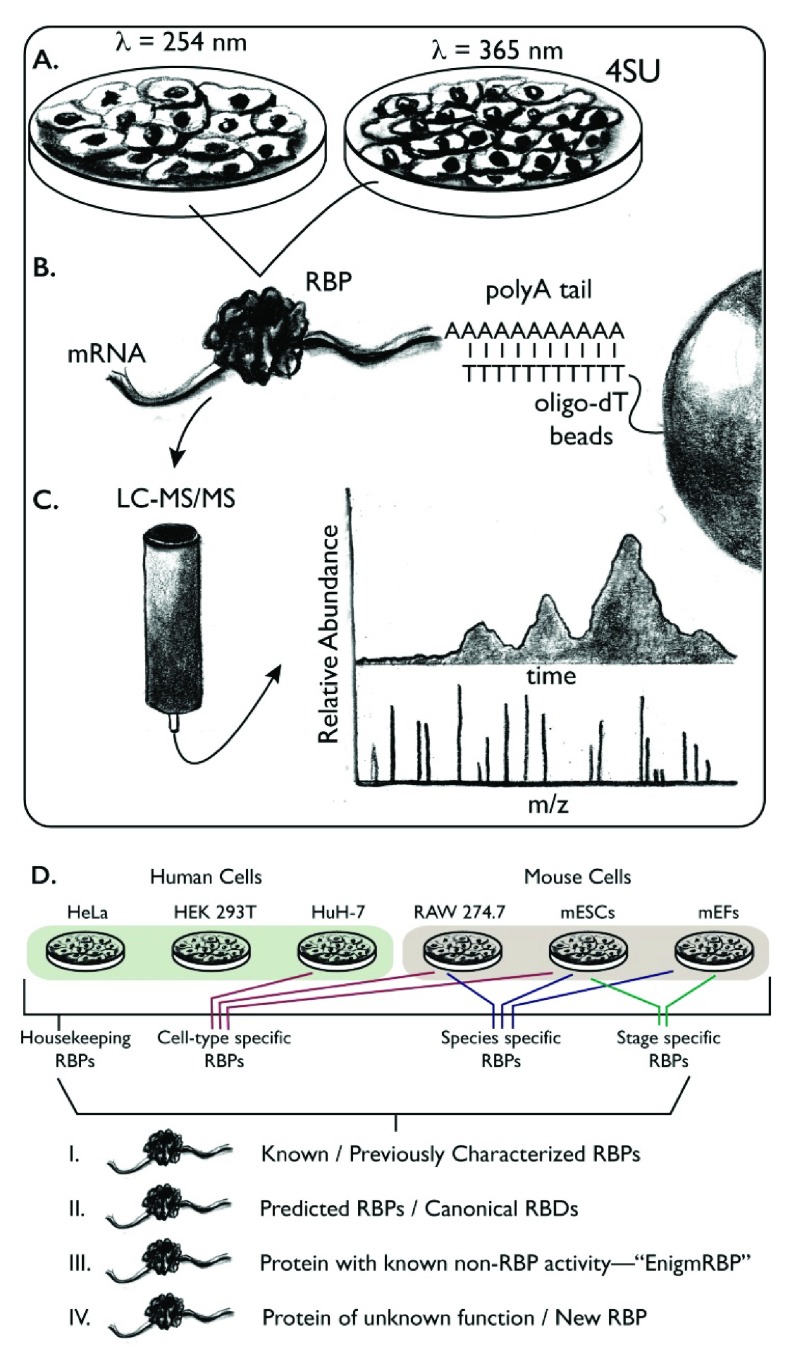
mRNA interactome capture. Schematic of mRNA interactome capture experiments. **A**. Samples are treated with ultraviolet (UV) light, either 254 nm to crosslink endogenous nucleotides to interacting proteins or 365 nm to crosslink exogenously added 4-thiouridine (4SU) to interacting proteins. **B**. Total mRNA is recovered from the sample using oligo-deoxythymidine (dT) resin, which base pairs with the polyadenosine (polyA) tail of mRNA. **C**. Following ribonuclease digestion, the proteins are separated and identified using quantitative liquid chromatography tandem mass spectrometry (LC-MS/MS). **D**. Summary of the outcomes. Some RNA-binding proteins (RBPs) are found in several samples, across diverse species. These proteins likely have housekeeping functions. Others are specific to unique cell types or are unique to a given species. Many well-studied RBPs are recovered by this approach, as well as many predicted RBPs with canonical domains such as RNA-recognition motif (RRM), KH, etc. New RBPs are also identified, which can be classified into groups that are entirely new or well-studied proteins where RBP-binding activity was unexpected (i.e. EnigmRBPs). Abbreviations: mEF, mouse embryonic fibroblast; mESC, mouse embryonic stem cells; RBD, RNA-binding domain.

Two independent teams, led by 1) Krijgsveld and Hentze and 2) Landthaler, simultaneously developed similar approaches for comprehensively mapping RBP–mRNA interactions in mammalian cell culture ( Figure 1) ^42, 43^. In brief, building upon a robust strategy first devised by Dreyfuss and colleagues in the 1980’s ^52^, proteins are crosslinked to RNA using ultraviolet (UV) light. Total mRNA is recovered using oligonucleotide-deoxythymidine (dT) conjugated resin under stringent wash conditions, then eluted by ribonuclease digestion. Covalently associated proteins are identified by high-resolution liquid chromatography tandem mass spectrometry (LC-MS/MS). While there are numerous details that distinguish the two studies in terms of crosslinking strategy, proteomics technique, and cell line investigated, both groups identified approximately 800 mRNA-interacting proteins, including many that were not previously known to bind RNA.

The study led by Krijgsveld and Hentze mapped the mRNA interactome in cultured HeLa cells ^43^. Two independent crosslinking strategies were used to diversify the type and number of crosslinks formed. In the first, short-wavelength (254 nm) UV light was used to crosslink endogenous nucleotides to their interacting proteins. In the second, a photoactivatable ribonucleoside (4-thiouridine, 4SU) was added to the culture media. When incorporated into RNA, this nucleotide analogue is capable of crosslinking to proteins with higher efficiency than endogenous nucleotides after activation by long-wavelength (365 nm) UV light ^53, 54^. The authors observed a remarkable 64% overlap in the protein content via both crosslinking strategies, with 24% specific to short-wavelength crosslinking and 12% specific to 4SU crosslinking. To assess the enrichment of proteins relative to control samples, the authors employed quantitative spectral and ion counting, which identified a total of 860 proteins at a false discovery rate (FDR) of 0.01. Shockingly, 315 of these proteins had not been shown or even predicted to bind to RNA, suggesting that a large fraction of protein-RNA biology remains unexplored. Many of the newly discovered RBPs contain serine/threonine kinase FAST and RAP domains, ZnF domains (which in fact have an extensive literature describing their RNA recognition properties ^55^), peptidyl-proline cis trans isomerase domains (PPI), or low-complexity repetitive sequences enriched in glycine, arginine, lysine, and/or tyrosine. Seventeen intermediary metabolism enzymes were recovered as well, consistent with a growing body of literature that demonstrates numerous metabolic enzymes also have functions in RNA biology, for example cytoplasmic aconitase ^56– 62^. Several of these were validated by immunoprecipitation of an eGFP-tagged candidate RBP from transfected cultured cells, annealing of texas red conjugated oligo-dT to the recovered RBP-mRNA complexes, and measuring the ratio of green and red fluorescence. One in ten of all enriched RBPs can be found in the online Mendelian inheritance in man (OMIM) database, a list of disease-related gene changes, suggesting many RBPs play critical roles in normal human physiology ^63, 64^.

**Figure 2.**
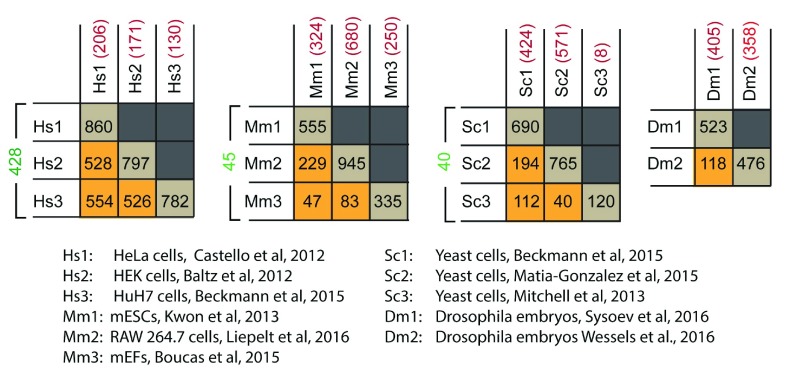
Comparison of multiple interactomes. Four tables are presented, comparing three human cell lines, three mouse cell lines, three yeast data sets, and two fly data sets, respectively. Below the tables is a key that identifies the source of the given interactome data set. The values in the tables represent the number of shared proteins in a pairwise comparison between the interactomes that intersect in the table. The diagonals (brown squares) show the total number of proteins collected in each given interactome. The orange squares show the number in common between two interactomes. The values in green to the left of each table are the number of proteins in common among the three interactomes compared, and the values in red are the number of proteins that are reported only in the specific interactome. Abbreviations: mEF, mouse embryonic fibroblast; mESC, mouse embryonic stem cell.

The study led by Landthaler also revealed many surprising findings ^42^. As above, the authors made use of 4SU crosslinking to covalently conjugate proteins to mRNA and recovered interacting proteins using oligo-dT resin. In contrast, this study, which employed HEK 293T cells, used stable isotope labeling by amino acids in cell culture (SILAC) for quantitative mass spectrometry and also made use of high-throughput sequencing to map the mRNA interactome footprint ^65^. Setting a threefold enrichment threshold in the SILAC data, the authors identified 505 unique proteins that showed enrichment in three replicates and a total of 797 that were enriched in at least one replicate. Of these, 245 were not previously annotated to be RBPs (~31%). The scope of RBP recovery is comfortingly congruent with the above study ^43^. Similarly, FAST kinase domain-containing proteins are abundant in the novel RBP category. Interestingly, the “winged-helix” family of transcription factors were also enriched, suggesting a possible dual role for these factors in both DNA and mRNA regulation ^66^. Perhaps most telling, there is significant overlap in identified RBPs in both studies, which identified 528 RBPs in common, despite being performed using different cell lines ( Figure 2). This suggests a high degree of reproducibility across methods, laboratories, and even different cell types.

To complement their proteomics studies, Landthaler and colleagues collected recovered mRNA and generated DNA libraries comparing crosslinked to non-crosslinked samples following proteinase K treatment ^42^. The libraries were sequenced and compared in order to identify thymidine to cytidine transitions that are characteristic of the position of crosslinking ^35, 67^. As such, the authors could identify individual bases in the population of total mRNA that were enriched for crosslinking to protein. The results define the interactome footprint, which reveals interesting features of mRNA that directly associate with protein. The most useful information stems from the interaction sites in the 3′ UTR, as crosslinking to ribosomes can cloud the interpretation of sites in the 5′ UTR and the coding sequence. Approximately 30% of all uridines in 3′ UTRs are readily converted to cytidine in the crosslinking analysis, suggesting that proteins heavily occupy the 3′ UTR on average. Moreover, sequence analysis reveals that nucleotides that flank the point of transition are less likely to include single nucleotide polymorphisms than other nucleotides, suggesting many crosslinked sites may be subject to evolutionary pressure.

Together, these studies broke the barrier to global mapping of the mRNA interactome ^42, 43^. Both studies are technical masterpieces, making use of cutting edge proteomics, informatics, and statistical tools to provide an atlas of all mRNA-binding proteins in mammalian cell culture. Though a huge breakthrough, the methods used in the studies are not without limitations. Most notably, the recovery of RBPs requires the presence of a polyadenosine tail on the mRNA. As such, pre-mRNAs that have not yet been adenylated, or stable deadenylated transcripts, will not be recovered by this approach. Proteins involved in pre-mRNA splicing or modification could be missed, as well as proteins that play a role in reversible translational silencing through deadenylation. Proteins that interact with RNA using interactions that are not compatible with short-wavelength crosslinking, or 4SU-mediated crosslinking, will likewise be missed. Finally, in some cases, the use of 4SU may alter the interactions present in the cell, leading to false negatives or false positives ^11^. Nevertheless, the volume of new RBPs discovered through these studies is likely to keep protein-RNA scientists busy for years to come, and the value of these unbiased studies should not be underestimated.

## The mRNA interactome in specialized cells

It is reasonable to expect that cells with different function and developmental potential will have inherently different mRNA interactomes. Following the lead of initial studies in HeLa and HEK 293T cells, recent work from several labs mapped the mRNA interactome in mouse embryonic stem cells (mESCs), mouse embryonic fibroblasts (MEFs), macrophages, and in the blood stages of the malarial parasite *Plasmodium falciparum*. These studies expand the mRNA interactome catalogue, enable the comparison of interactomes between multiple cell types, and have identified many additional RBPs.

In the first study, Kim and colleagues used short-wavelength UV crosslinking mRNA interactome capture in mESCs ^68^. They identified 555 proteins at an FDR of 0.01 in at least two of three biological replicates. Of these, 326 have homologs in both the previous studies using HeLa and HEK 293T cells, suggesting that these proteins are “core” mammalian RBPs that interact with mRNA in a wide variety of cells. Interestingly, 122 proteins appear only in the mESC data, and about half of them are enriched in mESCs relative to differentiated cells in RNA-seq data. Among the interesting findings from this work, the authors identified several WD40 proteins in the interactome ^69^, two well-studied ubiquitin ligases (Trim25 and Trim71) ^70^, and a variety of low-complexity putatively disordered proteins, many of which have been implicated in the formation of RNA granules as a result of phase separation ^71– 75^. Pathway analysis reveals many of the RBPs are downstream of c-myc, one of the Yamanaka factors that govern pluripotency ^76^. Together, the results reveal the mESC mRNA interactome and identify several new RBPs that could contribute to the maintenance of pluripotency.

A second study, by Dietrich, Reinhardt, and co-workers, investigated how the mRNA interactome of MEFs changes as a function of activating the DNA damage response with etoposide, a topoisomerase inhibitor ^46^. The authors identified 335 RBPs in untreated cells, 287 of which had been previously identified as RBPs in other studies. Upon treatment with etoposide, 44 RBPs changed in abundance: 30 showed reduced recovery and 14 showed increased recovery. To test whether there is a corresponding change in mRNA content, the authors performed RNA-seq on treated and untreated samples, which revealed a general correlation between changes in transcript abundance and the number of expected target mRNAs recognized by a given protein. Interestingly, the authors showed that four known RBP targets of p38 change upon etoposide treatment, including KHSRP, an RBP implicated in a variety of cell processes ^77^. The results reveal a dynamic mRNA interactome and identify a potential candidate RBP that could play a role in regulating cell cycle progression in response to DNA damage.

A recent study looked at the mRNA interactome in the blood stage of *P. falciparum* infection ^48^. *Plasmodium* is the causative agent of malaria, a prevalent insect-borne disease that affects 214 million people worldwide ^48^. Le Roch and colleagues used both computational prediction and mRNA interactome capture to identify RBPs from the *P. falciparum* genome ^48^. Their computational studies predicted approximately 1000 putative RBPs in the genome. Experimentally, they recovered 199 RBPs in at least two replicates of cultured trophozoite and schizont stage infected human type O+ erythrocytes. The most abundant and enriched RBPs from the *Plasmodium* genome include homologs of critical translation regulators, such as Musashi (HoMu), PfCelf2 (Bruno), PfAlba1, CITH, as well as several unstudied proteins containing predicted RRM, RAP, and DEAD-box domains. This work identifies several proteins that could play a role in the life cycle of this protozoan parasite, which could lead to new targets for anti-malarial interventions.

Most recently, Ostareck and Ostareck-Lederer used mRNA interactome capture to identify RBPs in murine RAW 274.7 macrophages that had been stimulated with lipopolysaccharide (LPS) compared to untreated controls ^49^. The innate immune system is tightly regulated at the post-transcriptional level and the stability of cytokine transcripts is regulated through RBP–mRNA interactions with destabilization sites in the 3′ UTR. Consistent with this, the authors identified 945 mRNA-interacting proteins in at least one of two replicates, 402 of which pass the statistical criteria used by the authors for inclusion in the interactome. These include 32 that appear to be unique to macrophages. Nineteen harbor no obvious RNA-binding motifs, including the HSP90 co-chaperone P23 and the hematopoietic-specific LYN 1 substrate adaptor protein HCLS1, which the authors subsequently demonstrated had RNA-binding activity using recombinant protein. The results also identify proteins that may be LPS-induction specific and as such may provide insights into post-transcriptional regulation of the innate immune response.

Taken in sum, we learn that many RBPs interact with mRNA in multiple cell types, suggesting that there are important “housekeeping” RBP–mRNA interactions that are fundamental to the physiology of all cells ( Figure 2). Nevertheless, specific differences are observed between different cell types, both in control conditions and when treated with agents that alter physiology, suggesting that RBP-mediated post-transcriptional regulation plays an important role in cell-type-specific responses. More work is needed to define the magnitude of their contribution and determine how they function at the mechanistic level.

## The mRNA interactome in model organisms

Model organisms are a valuable resource for understanding cell physiology, function, and differentiation across broad evolutionary diversity. Facile genetics coupled to a significant literature history enables the comparison of mRNA interactome data to extensive information derived from gene-specific studies. Following the publication of mRNA interactome capture technology ^42, 43^, there was naturally significant interest in applying the method to common model organisms. Now, several data sets for *Saccharomyces cerevisiae*, *C. elegans*, and *D. melanogaster* have emerged ^44, 45, 47, 50, 51^, enabling comparisons to the data sets collected using mammalian tissue culture and to each other.

One of the first efforts sought to identify the RBP content of haploid yeast grown under glucose deprivation to induce the stress response ^44^. Parker and colleagues used short-wavelength UV crosslinking followed by LC-MS/MS to identify 120 RBPs significantly enriched across two biological and five technical replicates. It is not immediately clear whether the difference in the overall number of RBPs compared to previous studies with mammalian cells is due to real biological differences between starved yeast and vertebrate cells or if variability in assay sensitivity and/or statistical criteria can account for the differences. As with studies in mammalian cells, many proteins recovered are well-characterized RBPs. Some of these are proteins that are known to bind to RNA but had not previously been implicated in mRNA binding. These include tRNA synthetases and tRNA-modifying enzymes, as well as ribosomal-processing proteins. It will be interesting to see if these proteins play dual roles in the cell, both in assembling the translational apparatus and in regulating gene expression. Such a role would not be unprecedented, as some ribosomal proteins have been shown to act as mRNA-specific translation regulators in several systems. As with previous studies, several “new” RBPs were identified, including a vacuolar ATPase subunit, several metabolic enzymes, low-complexity proteins, and protein kinases. In addition to this work, traditional crosslinking and immunoprecipitation (CLIP) studies ^37^ were performed on several RNA granule proteins identified in the interactome, defining sites of recognition in mRNAs.

A more recent study by Gerber and colleagues re-measured the yeast mRNA interactome and compared it to the *C. elegans* interactome defined under both normal and proapoptotic conditions ^47^. Using rich media cultures grown to mid-log phase, the authors identified 765 proteins in at least two of three independent replicates at an FDR of 0.01. About 70% of the yeast RBPs previously identified were confirmed in the new data set ^44^. Of the set of 561 predicted RBPs in the yeast genome, 205 were recovered in the mRNA interactome. Most of the proteins in the mRNA interactome (73%) were not predicted to bind RNA. Strikingly, 325 proteins encode metabolic enzymes, some of which have been implicated in RNA binding through previous gene-specific and interactome capture studies ^43, 56, 57, 78, 79^. It remains unclear whether the disparity between this study and the previous study of Parker and colleagues is due to different growth conditions or different assay sensitivity.

In the same study, the authors used interactome capture to identify 594 *C. elegans* mRNA-binding proteins ^47^. Proteins were considered significant if they appeared in at least two out of nine samples, using an FDR of 0.05. The first set of three samples comprised mixed-stage animals, which included all larval stages, adults, oocytes, and embryos. The second set was strictly L4 larval-stage animals, which produce sperm but have not yet begun to produce oocytes or embryos. The final set was L4 larval-stage animals treated with ethylnitrosourea (ENU), which induces apoptosis. Mixed-stage animals had 555 putative RBPs, while 39 proteins appeared exclusively in the L4 samples. The L4-enriched proteins include proteins required for spermatogenesis, including FBF-2 and ALG-4 ^21, 80^. Fifty-five proteins were shown to be enriched in ENU-treated samples and correspond to stress response proteins including EEF-1B.1 and EEF-1B.2, as well as translation initiation factors EGL-45, EIF-3, EIF-6, and IFE-2, and CLU-1. As with yeast, about 40% of the total mRNA interactome consists of proteins annotated as metabolic enzymes. Similar to other studies, low-complexity domains were also enriched. Comparison of the new yeast mRNA interactome with the *C. elegans* interactome revealed more than half of the *C. elegans* RBPs had a corresponding homolog in the yeast data set. This suggests extensive conservation of RBP function throughout evolution.

A third study, from Krisjgsveld, Hentze, and colleagues, measured the yeast mRNA interactome a third time and compared it to the RBP content of human hepatocytic HuH-7 cells ^45^. In this study, yeast RBPs were captured using 4SU crosslinking, while HuH-7 cells were analyzed through both conventional crosslinking and 4SU crosslinking, to facilitate comparisons to RBPs identified in their original study using HeLa cells. Under stringent selection criteria across three biological replicates, the authors identified 678 yeast mRNA-interacting proteins and 729 HuH-7 mRNA-interacting proteins at an FDR of 0.01. Of the 120 RBPs identified by Parker and co-workers, 108 are confirmed in this study ^44^. A direct comparison between this data set and that of Gerber and colleagues was not made ^47^, as the two studies were published within two weeks of each other. However, the scope of the recovered RBPs was similar in magnitude (678 vs. 765, respectively), as well as the fact that many recovered RBPs are not annotated as RNA binding but have well-studied function in metabolism or other cell processes. The authors of the current study define such proteins as “EnigmRBPs”.

The HuH-7 data identified 109 RBPs that were not present in previously published data sets using HeLa and HEK 293T cells, suggesting that these proteins are specific to hepatocytes or interact with RNA differentially between cell types and/or culture conditions. Consistent with previous findings, most of the proteins are recovered from all three cell types, suggesting a possible “housekeeping” function for many RBPs. To investigate an EnigmRNP in more detail, the authors evaluated the RNA-recognition properties of the mitochondrial hydroxysteroid dehydrogenase (HSD17B10). This enzyme is a polyfunctional protein that has been reported to play an important role in metabolizing steroids, in isoleucine catabolism, and, to a lesser extent, acting as an alcohol dehydrogenase ^81, 82^. Surprisingly, it also appears to be a subunit of a multi-protein complex with RNAse P activity, trimming 5′-leader sequences from precursor tRNA in the mitochondria ^83^. Consistent with this role, iCLIP experiments ^84^ demonstrated that HSD17B10 indeed binds preferentially to the 5′-leader sequence of some but not all mitochondrial tRNAs and that a disease-causing mutant variant of the protein reduces its ability to bind to RNA. These results suggest that the tRNA-binding activity of HSD17B10 is important and that disruption of this activity, rather than its metabolic activities, might be at the heart of its dysfunction in human cardiomyopathy. It is not yet clear how this enzyme, which appears to be primarily involved in mitochondrial tRNA processing, also interacts with polyadenylated mRNA.

The final two mRNA interactome studies sought to define how the interactome changes during the maternal-to-zygotic transition (MZT) of *Drosophila* embryos ^50, 51^. In flies, and most metazoans, maternally supplied mRNAs and proteins are crucial for early patterning events ^85^. At the MZT, maternal mRNAs are replaced with transcripts encoded by the embryonic genome in a process that likely requires coordinated turnover of maternal RNAs and a mechanism to distinguish newly synthesized mRNAs from those stored in the oocyte cytoplasm ^86^. RBPs are critical for controlling maternal mRNA expression, so there is great interest in understanding how the mRNA interactome changes during the MZT ^86^.

In the study by Ohler, Landthaler, and colleagues, 476 “high confidence” RBPs were identified in both conventional and 4SU-mediated crosslinked samples in mRNA interactome capture experiments using *Drosophila* embryos 0–2 hours post-fertilization ^51^. As with previous studies of yeast and *C. elegans*, many of the recovered proteins (164) have clear homologs in the mammalian interactome capture experiments. The rest are either fly specific (47) or are preferentially expressed in oocytes/embryos. Of these proteins, 57% harbor a canonical RNA-binding domain, while the rest are similar to the new RNA-binding domain classes identified in previous studies ^42, 43^, including WD40 repeats and protein kinases. Interestingly, mRNAs that encode RBPs identified through interactome capture tend to be found in earlier embryos, while mRNAs that encode transcription factors are more abundant in later embryos. This is consistent with the hypothesis that RBPs in early embryos are enriched in factors that control the timing and expression of maternal RNAs (i.e. post-transcriptional regulators), and their influence is less substantial compared to transcription factors in older embryos, following the MZT.

In a competing study by Ephrussi and colleagues, the mRNA interactome was determined using conventional crosslinking from 0–1 hour post-fertilization embryos as a pre-MZT sample, and 4.5–5.5 hours post-fertilization embryos as a post-MZT sample ^50^. In total, 523 proteins were identified between the two samples at an FDR of 0.01. Of these, 236 have not been shown or predicted to bind to RNA previously, although some of these have homologs that were identified in previous mRNA interactome capture studies from other species. Of the fly embryo mRNA interactome set, 236 RBP-encoding genes give lethal or sterile phenotypes upon mutation, many of which are also annotated as having embryonic development phenotypes. Fifteen of these did not have a previously assigned molecular function, demonstrating the value of unbiased mRNA capture in the identification of new RBPs with important biological phenotypes in model. In an interesting finding, several cytoskeletal regulators were identified as RNA binding, including CycB and EB1, proteins critical to mitotic division ^87, 88^. The developmentally staged interactome data also revealed surprises about the dynamics of mRNA binding. Only 12 are identified as being enriched in one sample relative to the other at an FDR of 0.01. This expands to 116 proteins at a more lenient FDR of 0.1. Of these, 27 show significant protein abundance changes as a function of developmental stage. The only enriched gene ontology (GO) term for these 27 is “mRNA splicing”, suggesting that developmentally timed splicing isoform ratio changes might be important to the MZT, although more work is needed to flesh out exactly how these proteins contribute to splicing regulation and how important they are to the MZT. Nevertheless, it is clear that much can be learned from mRNA interactome capture experiments, including similarities across species, responses to stress or growth conditions, developmentally programmed changes, and more.

In an effort to facilitate such comparisons, I have prepared tables that identify the overlap between RBPs recovered in various interactome measurements ( Figure 2). This analysis shows 428 RBPs found in common among human-derived HeLa, HEK 293T, and HuH-7 cells ^42, 43, 45^; 45 RBPs in common among mESCs, MEFs, and murine RAW 264.7 macrophage cells ^46, 49, 68^; 40 RBPs in common among the three sets of data covering ** yeast ^44, 45, 47^; and 118 RBPs in common between the two fly data sets ^50, 51^. Comparing the yeast data, nearly all of the RBPs identified in the original study by Mitchell *et al*. have been confirmed by at least one other study, with only eight RBPs identified uniquely by the Mitchell *et al*. study ^44^. This suggests that these RBPs are very high confidence, consistent with the stringent requirements used in their identification. One surprising result from this comparison is that mESCs appear to have more RBPs in common with RAW 264.7 cells (194 RBPs) than with MEFs (112 RBPs). This result may be an artefact of non-saturation of the MEF mRNA interactome, which is smaller than the other two (MEFs = 335, mESCs = 555, RAW 264.7 = 945 RBPs), or overinterpretation of the RAW 264.7 interactome, which was not filtered for specificity, LPS responsiveness, or statistical significance in my comparisons for the sake of convenience. Overall, a general note of caution is warranted in the direct comparison of interactome lists, as differences in mass spectrometry quantitation methods, crosslinking approach, and statistical thresholding may present the appearance of “uniqueness” in a given data set, when in fact a deeper dive into the data or higher throughput may reveal an increasing number of similarities. Ultimately, comparison of the data reveals several RBPs that are shared across numerous cell types, but, as always, it is not safe to assume that the absence of an RBP from a given data set is proof that it is not active in that cell type.

## Next steps

Now that mRNA interactome capture has been adopted by several groups and applied to several types of samples, it has emerged as a powerful approach to identify novel RBPs in a wide range of situations. The method is already being used to investigate important biological issues, such as embryonic patterning ^50, 51^, DNA damage response ^46, 47^, and starvation ^44^. In essence, any system where post-transcriptional regulation is biologically important is a likely source for new studies using the approach. For example, in the brain, post-transcriptional regulation is required for localizing mRNAs to synapses, for localized translation, and for the regulation of alternative splicing ^89, 90^. It will be interesting to see how the mRNA interactome changes in neurons and other cells of the brain in response to insults such as oxidative stress, neurodegenerative disease, in response to learned behaviors, *et cetera*. It would also be interesting to see how the mRNA interactome changes in inherited disease states, as RBPs are being linked to an increasing number of genetically transmitted maladies ^43^. Ultimately, through such studies, I expect the pace of new RBP discovery to remain high for several years.

The next obvious goal following mRNA interactome capture is to pair each RBP with the suite of mRNAs that it recognizes, as was recently done with the EnigmRNP HSD17B10 ^45^. While interactome capture can identify new RBPs, it falls short of mapping pairwise interactions between all RBPs and their mRNA targets. Ultimately, this information will be necessary to complete the wiring diagram and develop hypotheses for how each RBP functions in the cell. This will require the development of new technology, or the application of existing single gene methods such as CLIP and PAR-CLIP at much higher throughput ^35, 36, 84, 91^, which will require a dramatic increase in resources and effort. It is not clear to me that such approaches will have the necessary throughput to complete the RBP-mRNA wiring diagram on a protein-by-protein basis. A new study, published while this article was in review, presents a mass spectrometry-driven approach to identify RBP-binding sites within mRNAs in higher throughput and may provide the necessary tools to begin making such connections on a grander scale ^92^.

Finally, it will be necessary to develop tools to connect mRNA interactome measurements with functional assays to determine how important each interaction is to the physiology of the cell or animal. Lessons from the gene regulatory network literature suggest that binding is not always a good proxy for function ^93– 97^. While there is no clear high-throughput approach to accomplish this goal at this time, it is certain that numerous labs are interested in finding a solution to this particular problem, and as such there is hope that a full wiring diagram of post-transcriptional regulation will be completed in our lifetimes.

## Abbreviations

4SU, 4-thiouridine; CLIP, crosslinking and immunoprecipitation; dT, deoxythymidine; ENU, ethylnitrosourea; FDR, false discovery rate; LC-MS/MS, liquid chromatography tandem mass spectrometry; LPS, lipopolysaccharide; Mago, Mago nashi; MEF, mouse embryonic fibroblast; mESC, mouse embryonic stem cell; mRNA, messenger RNA; MZT, maternal-to-zygotic transition; Puf, Pumilio-FBF; RBP, RNA-binding protein; RRM, RNA-recognition motif; SILAC, stable isotope labeling by amino acids in cell culture; UV, ultraviolet, UTR, untranslated region.
